# Glypican 3-Targeted Therapy in Hepatocellular Carcinoma

**DOI:** 10.3390/cancers11091339

**Published:** 2019-09-10

**Authors:** Takahiro Nishida, Hiroaki Kataoka

**Affiliations:** 1 Section of Oncopathology and Regenerative Biology, Department of Pathology, Faculty of Medicine, University of Miyazaki, 5200 Kihara, Kiyotake, Miyazaki 889-1692, Japan; 2 Division of Gastrointestinal, Endocrine and Pediatric Surgery, Department of Surgery, University of Miyazaki Faculty of Medicine, 5200 Kihara, Kiyotake, Miyazaki 889-1692, Japan

**Keywords:** hepatocellular carcinoma, glypican-3, codrituzumab, T cell-redirecting antibody, CAR-T

## Abstract

Glypican-3 (GPC3) is an oncofetal glycoprotein attached to the cell membrane by a glycophosphatidylinositol anchor. GPC3 is overexpressed in some kinds of tumors, particularly hepatocellular carcinoma (HCC). The prognostic significance of serum GPC3 levels and GPC3 immunoreactivity in tumor cells has been defined in patients with HCC. In addition to its usefulness as a biomarker, GPC3 has attracted attention as a novel therapeutic target molecule, and clinical trials targeting GPC3 are in progress. The major mechanism of anti-GPC3 antibody (GPC3Ab) against cancer cells is antibody-dependent cellular cytotoxicity and/or complement-dependent cytotoxicity. Since GPC3Ab is associated with immune responses, a combination of protocols with immune checkpoint inhibitors has also been investigated. Moreover, some innovative approaches for GPC3-targeting therapy have emerged in recent years. This review introduces the results of recent clinical trials targeting GPC3 in HCC and summarizes the latest knowledge regarding the role of GPC3 in HCC progression and clinical application targeting GPC3.

## 1. Introduction

Primary liver cancer is the sixth most common malignant neoplasm and the second most common cause of cancer-related death worldwide [1]. Hepatocellular carcinoma (HCC) is the most common form of primary liver cancers and over 80% of patients with HCC have liver cirrhosis associated with hepatitis B and C virus infection, alcohol abuse, or non-alcoholic fatty liver disease [2,3]. Because only 40% of HCC patients are detected at an early stage [4], there is a critical need for the development of innovative detection systems and treatments for HCC with poor prognosis.

The multikinase inhibitor sorafenib has been used as a first line of chemotherapy for advanced HCC for the last 10 years [5], but recently, new molecular targeting drugs have emerged and their efficacies in clinical trials are under extensive investigation. The results of randomized phase III clinical trials have been reported [6,7,8,9]. Lenvatinib, a multikinase inhibitor that effectively inhibits vascular endothelial growth factor receptors (VEGFR) 1-3, fibroblast growth factor (FGF) receptors 1–4, platelet-derived growth factor receptor α, and RET, and KIT, showed similar efficacy to sorafenib in median overall survival (OS) as a first-line treatment for unresectable HCC (lenvatinib vs. sorafenib; 13.6 months vs. 12.3 months, respectively, with hazard ratio [HR] 0.92 and 95% confidence interval [CI] 0.79–1.06) in the REFLECT study (NCT01761266) [6]. Cabozantinib, a tyrosine kinase inhibitor of VEGFR 1–3, MET, and AXL, is intended for patients with previously treated advanced HCC. It resulted in a longer median OS (cabozantinib vs. placebo, 10.2 months vs. 8 months, respectively, with HR 0.76 and 95% CI 0.63–0.92, *p* = 0.0049). Median progression-free survival (PFS) was also improved compared to the placebo group (cabozantinib vs. placebo; 5.2 months vs. 1.9 months, respectively, with HR 0.44, 95% CI 0.36–0.52, *p* < 0.001) in the CELESTIAL study (NCT01908426) [7]. Ramucirumab, a VEGFR 2 antagonist, also showed a positive outcome as a second-line therapy for advanced HCC in the REACH and REACH-2 studies (NCT01140347 and NCT02435433) [8,9].

Immunotherapies may provide significant breakthroughs in cancer treatment. Immunotherapy can be divided into immune-brake molecular targeting therapy and immune-accelerator therapy. For the immune-brake molecular targeting therapies, immune checkpoint inhibitors, such as anti-programmed cell death protein-1 (PD-1), anti-PD-1 ligand (PD-L1), and anti-cytotoxic T lymphocyte-associated protein-4 (CTLA-4) antibodies, are well established [10,11]. On the other hand, cancer vaccine therapy and chimeric antigen receptor-T cells (CAR-T) therapy are immune-accelerator therapies in which a target molecule expressed by cancer cells is crucial [12]. As a representative molecule specifically expressed by cancer cells, glypican-3 (GPC3) in HCC has attracted attention.

## 2. GPC3: Structure, Expression, and Functions

In 1988, Filmus et al. isolated a cDNA clone that corresponded to a developmentally regulated transcript in a cell line from rat small intestine [13]. The cDNA clone was named OCI-5. The human *OCI-5* gene encodes a protein highly homologous to the glypican family and is now widely known as GPC3 [14].

**Figure 1:**
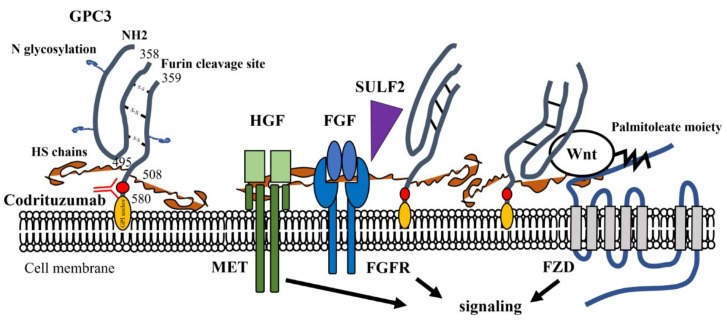
Structure of the glypican-3 (GPC3) molecule and possible involvement GPC3 in progression of HCC. The core protein consists of 580 amino acids. Two heparan sulfate (HS) side chains are attached near the C-terminal portion. Codrituzumab (GC33) recognizes the epitope near the glycosyl-phosphatidylinositol (GPI) anchor. Growth factors such as Wnt, fibroblast growth factor (FGF), and hepatocyte growth factor (HGF) can be complexed with HS side chains. In Wnt signaling, GPC3 core protein functions with Frizzled receptor (FZD) as a co-receptor. Sulfatase 2 (SULF2) is known to release FGF to transduce signals through its specific receptor. MET: receptor of HGF; FGFR: fibroblast growth factor receptor; FZD: Frizzled receptor.

GPC3 is an oncofetal heparan sulfate (HS) glycoprotein attached to the cell membrane by a glycophosphatidylinositol (GPI) anchor [15,16]. The GPC3 core protein consists of 580 amino acids and is 70 kDa in size. Two HS side chains are attached near the C-terminal portion. The single-chain GPC3 is processed by furin at the Arg^358^-Cys^359^ bond to generate the mature GPC3 consisting of a 40-kDa N-terminal subunit and a 30-kDa C-terminal subunit linked by disulfide bonds [17] (Figure 1). Various forms of GPC3 can be detected in culture supernatant of GPC3-expressing cells and in serum, indicating that proteolytic cleavages of the extracellular portion of GPC3 may occur [18,19,20].

The *GPC3* gene is located on the X chromosome (Xq26.2). GPC3 is believed to play a crucial regulatory role in cellular proliferation in embryonic mesodermal tissues since deletion of the *GPC3* gene leads to the development of gigantism/overgrowth syndrome known as Simpson–Golabi–Behmel syndrome (SGBS) [21,22,23,24,25]. Mechanistically, GPC3 is likely involved in the regulations of the signaling pathways of Wnt, hedgehog, bone morphogenic protein, and FGF. In this way, it controls cell growth and apoptosis in certain cell types during development [26,27,28,29]. GPC3 is widely expressed in the placenta, as well as the liver, lungs, and kidneys of the embryo. In contrast, it is hardly detectable in most organs in adults [30]. This biological downregulation in adult tissues may be explained by DNA methylation within the *GPC3* promoter region [31,32,33].

## 3. GPC3 and Tumor Progression

The expression of GPC3 has been reported in various tumors, such as HCC, lung squamous cell carcinoma (SqCC), gastric carcinoma, ovarian carcinoma, melanomas, and pediatric embryonal tumors. Among them, the expression is particularly high in HCC [34,35,36,37,38,39]. In HCC cells, evidence suggests that GPC3 is involved in Wnt/β-catenin signaling and enhances proliferation of the cells [40] (Figure 1). GPC3 core protein interacts with the Wnt receptor Frizzled (FZD) [41], and a recent study has revealed that GPC3 core protein functions as a co-receptor for Wnt to promote the Wnt/β-catenin signaling in HCC cells [42]. Upregulated GPC3 accelerates the progression of lung SqCC cells in a Wnt/β-catenin-dependent manner [43]. On the other hand, sulfatase 2 (SULF2), an enzyme with 6-O-desulfatase activity on HS proteoglycans, is upregulated in HCC cells leading to enhanced release of the heparin-binding growth factors such as FGF and HGF attached to the HS sidechains of GPC3 [44,45], which in turn activates the signaling pathways mediated by their specific receptors [44,45,46,47]. Wnt can also attach to HS; thus, SULF2 may also enhance the Wnt signaling [44,48]. In contrast, soluble GPC3 is known to suppress the proliferation of cancer cells [49,50]. Cell surface GPC3 is thought to be released by the cleavage of the GPI anchor by sheddase, such as phospholipase D [51]. Although notum was once considered as a sheddase of GPC3 [52], it is now established that notum is a deacetylase and cleaves the palmitoleate moiety of Wnt attached to the HS chain but not the GPI anchor of GPC3, and inhibits the binding of Wnt to FZD [53]. Taken together, GPC3 may be a crucial molecule in cancer cell biology, and the cell surface GPI-anchored GPC3 might serve as a reservoir and cofactor for paracrine or autocrine growth factors to efficiently transduce their outside-in signaling. On the other hand, shedding of GPC3 from the cell surface likely disturbs these signaling pathways. Therefore, further studies to understand the molecular mechanism and regulation of GPC3 shedding from cancer cells’ surfaces will be required.

Micro RNAs (miRNAs) and long noncoding RNA (lncRNA) have recently emerged as regulators of gene expression in cancer cells [54,55]. Several miRNAs and lncRNAs have been reported to promote or suppress the GPC3/Wnt/β-catenin axis in HCC [56,57,58,59,60]. Further investigations should be conducted to better understand miRNAs, lncRNA, and related axes critical to GPC3 expression.

## 4. Clinical Trials of GPC3 Targeting Therapy

### 4.1. GPC3-Targeted Antibody Therapy

**Figure 2:**
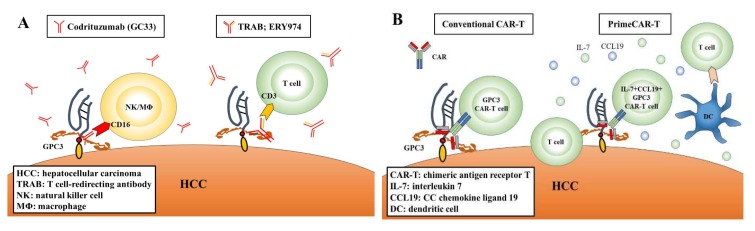
Schema of anti-tumor effect in immunotherapy. (**A**) Codrituzumab (GC33) induces ADCC by effector cells. TRAB (ERY974) further activates T cells. (**B**) Genetically engineered T cells navigated to glypican-3 (GPC3) are reinfused into patients in conventional CAR-T therapy. PrimeCAR-T has enhanced infiltration, accumulation, and survival capabilities in solid tumors.

GC33 (codrituzumab), a recombinant humanized monoclonal antibody, binds to the juxtamembrane domain of GPC3 with high affinity. GC33 induces antibody-dependent cellular cytotoxicity (ADCC) and/or complement-dependent cell cytotoxicity (CDC) and inhibits tumor growth, a process in which natural killer (NK) cells play a major role as effector cells for ADCC [61,62] (Figure 2A). In phase I trials in the United States and Japan, codrituzumab was well tolerated and showed antitumor effects in patients with HCC [63,64]. However, it could not show a benefit in a randomized phase II trial with 121 HCC patients [65]. In the phase II trial of codrituzumab vs. placebo, median PFS was 2.6 months vs. 1.5 months, respectively, with a hazard ratio [HR] 0.97 and *p* = 0.87, and median OS was 8.7 months vs. 10 months, respectively, with HR 0.96 and *p* = 0.82 [65]. Nevertheless, sub-analysis of the results of the phase II trial and another study indicated that codrituzumab showed prognostic merit for HCC patients with a high immunogenicity related to ADCC and a high expression of GPC3 [65,66].

Recently, the introduction of genetically modified bifunctional antibodies has attracted attention to them as novel immunotherapeutic drugs. Newly developed ERY974, a humanized IgG4 bispecific T cell-redirecting antibody (TRAB), has two different heavy chains and a common light chain, and the heavy chains recognize GPC3 or CD3 [67] (Figure 2A). In vitro, ERY 974 activated T cells in a GPC3-dependent manner and showed anti-tumor effects on various solid cancer cells expressing GPC3, including HCC [68]. Considering the high efficacy of GPC3-targeted TRAB, this approach may be valid for non-HCC solid carcinomas that express low but distinct levels of GPC3. Based on the pre-clinical research data, a phase I trial is now ongoing for patients with GPC3-positive solid carcinoma (NCT02748837) (Table 1).

The combination of an anti-GPC3 antibody and an immune checkpoint targeting antibody is also an attractive protocol for the treatment of GPC3-expressing cancers. In a mouse model of a GPC3-overexpressing liver tumor, anti-mouse GPC3 monoclonal antibody showed more potent anti-tumor activity when combined with anti-mouse PD-L1 antibody [69]. Indeed, a phase I clinical trial of the combination treatment is currently in progress, and a recent report indicated that codrituzumab plus atezolizumab combination therapy was well tolerated and showed antitumor activity in advanced, previously treated, and GPC3 overexpressed HCC [70] (Table 1).

### 4.2. Vaccine Therapy

In peptide vaccine therapy for cancer, dendritic cells (DC) recognize HLA class I-restricted peptides derived from specific molecules expressed on cancer cells and specifically induce cytotoxic T lymphocytes (CTL) against them. So far, clinical trials using HLA-A24- and HLA-A2-restricted GPC3-derived peptides have been performed (Table 1). In phase I trials for patients with advanced HCC, GPC3 vaccination was well tolerated and the vaccine induced a high rate of GPC3-specific CTL responses [71]. In phase II trials, HCC patients who underwent surgery or radiofrequency ablation (RFA) were enrolled, and one- and two-year recurrence rates were set as the primary endpoints. The vaccination did not show a benefit, and the one-year recurrence rates in patients with the initial treatment + vaccination vs. the initial treatment alone were 28.6% and 54.3%, respectively (*p* = 0.346), and the two-year recurrence rates were 39.4% and 54.5%, respectively (*p* = 0.983) [72]. On the other hand, similar to codrituzumab, when the analysis was limited to GPC3-expressing HCC, the recurrence rate in the first treatment + vaccination group was significantly lower compared to that in the group of initial treatment only: 24% vs. 48%, *p* = 0.047 for one-year recurrence rates, and 52.4% vs. 61.9%, p = 0.387 for two-year recurrence rates) [72].

### 4.3. CAR-T Therapy

CAR-T cells are generated from the patient-derived T cell. After genetically engineered T cells are expanded, they are reinfused into the patients (Figure 2B). This therapy is highly effective for relapsed or non-remitting young patients of B-cell acute lymphocytic leukemia and adult diffuse large B-cell lymphoma [73,74]. In CAR-T therapy in these hematological malignancies, CAR-T cells are mainly activated through CD19 antigens on the target cells.

To develop CAR-T therapy of solid cancers, antigen molecules specifically and abundantly expressed on the cancer cell surface need to be explored. In this regard, GPC3 may serve as a promising cell surface antigen for the generation of CAR-T cells targeting HCC cells. According to https://clinicaltrials.gov/ (as of 27 June 2019), 11 GPC3-targeted CAR-T therapies are registered. Among them, 9 studies provide information regarding the current status, in which two trials had been completed, three are currently in progress, and four are in preparation (Table 2).

The results of a phase I study (NCT02395250) with 13 Chinese patients with refractory or relapsed GPC3-positive HCC was reported [75]. The patients tolerated the procedure and preliminary analysis suggested certain efficacy when lymphodepleting conditioning was applied along with GPC3 CAR-T [75]. The validation studies for the combination of GPC3 CAR-T therapy with a molecular targeting drug or immune checkpoint inhibitor were also performed in mouse models and showed promising results [76,77]. Recently, methods were altered by Adachi et al. [78] to significantly improve the efficiency of CAR-T, namely proliferation-inducing and migration-enhancing chimeric antigen receptor-T (PrimeCAR-T) therapy. They developed CAR-T cells that express interleukin 7 (IL-7) and chemokine (C-C motif) ligand 19 (CCL19) [78] (Figure 2B). IL-7 stimulates T cell survival and proliferation, and CCL19 stimulates T cell and dendritic cell migration, and in lymphoid organs, IL-7 and CCL19 produced by T-zone fibroblastic reticular cells are essential for T-zone formation and maintenance [78]. Therefore, PrimeCAR-T mimics T zone reticular fibroblast function to mobilize T cells and DC to tumor tissue [78]. As the PrimeCAR-T method significantly improved the anti-tumor effects compared with conventional CAR-T in mouse models [78], clinical trials of the PrimeCAR-T therapy for solid cancers are anticipated and its application to the GPC3-targeted therapy may be a very promising approach.

## 5. Detection of GPC3 In Vivo

Serum GPC3 levels and GPC3-immunohistochemistry (GPC3-IHC) have proven useful as biomarkers and prognostic factors for HCC patients [79,80,81,82,83,84]. For serum GPC3 levels, a several sandwich enzyme-linked immunosorbent assay (ELISA) have been reported, using antibodies recognizing full-length GPC3, a N-terminal subunit of GPC3, or C-terminal subunits of GPC3 [18,79,80,83]. Although meta-analysis of the literature indicated that serum GPC3 is higher in HCC patients than normal subjects, the reported values differed considerably between the studies, probably due to different antibody epitopes in each ELISA setting and heterogeneity of molecular forms of serum GPC3 [83,85,86]. Some studies showed the serum GPC3 levels tended to be higher in the order of HCC, liver cirrhosis, and chronic hepatitis patients [85], but its diagnostic utility is still a matter of debate. Haruyama et al. reported that a sandwich ELISA system that recognized the N-terminal subunit of GPC3 was highly sensitive [79]. With this assay, about 60% of HCC cases showed abnormally high preoperative serum GPC3N levels (>mean GPC3 + 2 SD of healthy controls) and the high preoperative levels were significantly associated with shorter OS and disease-free survival after hepatectomy [79]. On the other hand, evidence obtained by GPC3-IHC studies revealed acceptable sensitivity and specificity for diagnostic purposes of HCC. While GPC3 was hardly detectable in normal liver and chronic hepatitis, more than 70% of HCC cases showed positive immunoreactivity [18,79,81,82]. Dysplastic regenerative nodules in cirrhotic liver also showed focal and weak immunoreactivity [18,83,87]. Recently, novel approaches have been developed for in vivo detection of GPC3-expressing HCC in both mouse models and human patients. In conventional positron emission tomography (PET) imaging for tumor detection, 2-^18^F-fluoro-2-deoxy-D-glucose is generally used as a probe. Recently, novel GPC3-specific probes, such as ^18^F-labeled GPC3-targeting peptides [88,89] and ^124^I-labeled codrituzumab [90], have been synthesized, and PET imaging with these novel probes detected GPC3-expressing tumors with higher sensitivity compared to conventional approaches [88,89,90]. In magnetic resonance imaging, a GPC3-targeted aptamer was directly or indirectly combined with an iron oxide contrast agent, an ultrafine superparamagnetic iron oxide [91,92]. These GPC3-specific imaging methods could provide an accurate assessment of tumor responses to GPC3-targeted therapy and, more importantly, allow the early detection of GPC3-expressing HCC suitable for the GPC3-targeted therapy in the future.

## 6. Conclusions and Future Perspectives

HCC is a deadly disease, and researchers are searching for innovative strategies to detect and treat this extremely malignant tumor. GPC3 is a unique molecule specifically expressed on the surface of HCC cells such that the level of expression may predict the patient’s prognosis [79,80,81,82,83,84]. These findings provide rationales for the use of this molecule in diagnosis, clinical management and molecular targeting therapy in HCC. These GPC3-targeting strategies can also be applied to other solid carcinomas that aberrantly express GPC3. Various preclinical and clinical studies are underway using innovative GPC3-targeting therapies, such as the combined treatment of anti-GPC3 antibody with another molecular targeting drug, GPC3-targeted TRAB, GPC3 peptide vaccination, and GPC3 CAR-T therapies. However, many problems remain with regard to their efficacy and dynamics. To develop a safer and more effective GPC3-targeted treatment, it is still necessary to clarify GPC3’s molecular functions, transcriptional regulation, and post-transcriptional modification, including shedding from the cell surface.

## Figures and Tables

**Table 1:** Clinical trials of GPC3-targeted antibody therapy and vaccine therapy.

**Study Title**	**Phase**	**Trial No.**	**Conditions**	**Outcomes**	**Reference**
Molecular targeting therapy					
A phase I study of GC33 in advanced or metastatic liver cancer (hepatocellular carcinoma)	I	NCT 00746317	Advanced or metastatic HCC	GC33 was well tolerated. GPC3 expression in HCC may be associated with the clinical benefit to GC33.	Zhu et al. 2013 [63]
Phase I study of GC33 in patients with advanced HCC	I	JapicCTI 101255	Japanese patients with advanced HCC	GC33 was well tolerated. The correlation between antitumor activity and GPC3 expression was not clear.	Ikeda et al. 2014 [64]
A study of RO5137382 (GC33) in patients with advanced or metastatic HCC	II	NCT 01507168	Patients with advanced HCC who had failed prior systemic therapy	Codrituzumab did not show any clinical benefit. A high dose of codrituzumab or a high GPC3 level or its mediator CD16 may improve outcome.	Abou-Alfa et al. 2016 [65]
Vaccines therapy					
Phase I clinical study of glypican-3 peptide vaccine in patients with advanced HCC	I	UMIN 000001395	Advanced HCC patients	GPC3 vaccination was well tolerated. Peptide-specific CTL frequency may be a predictive marker of OS.	Sawada et al. 2012 [71]
A phase II study of GPC3 peptide vaccine as adjuvant treatment for HCC after surgical resection or radiofrequency ablation (RFS)	II	UMIN 000002614	Patients with initial HCC who had undergone surgery or radiofrequency ablation	GPC3 vaccination did not have longer RFS or OS. Vaccination in patients with GPC3-positive tumors improved 1-y recurrence rates.	Sawada et al. 2016 [72]
**Ongoing GPC3-Targeted Antibody Therapy Trials**	**Phase**	**Trial No.**	**Conditions**	**Status**	**Country**
A study of ERY974 in patient with advanced solid tumors	I	NCT 02748837	GPC3-positive advanced solid tumors	Active, not recruiting	United States
A phase I study of codrituzumab, in combination with atezolizumab in patients with HCC	I	JapicCTI 163325	Locally advanced or metastatic HCC in which GPC3 is expressed by IHC	Active, not recruiting	Japan, Taiwan

CTL: cytotoxic T lymphocyte; OS: overall survival; RFS: recurrence-free survival; IHC: immunohistochemistry.

**Table 2:** Ongoing or planned GPC3-targeted chimeric antigen receptor (CAR)-T therapy studies in HCC.

Study Title	Phase	Trial No.	Conditions	Status	Country
Anti-GPC3 CAR-T for treating patients with advanced HCC	I	NCT 02395250	Non-diffuse HCC with the presence of extrahepatic metastasis or portal vein vascular invasion. GPC3 is expressed by IHC.	Completed	China
CAR-T cell immunotherapy for HCC targeting GPC3	I & II	NCT 02723942	Non-diffuse HCC, no extrahepatic metastasis or portal vein vascular invasion. GPC3 high expression HCC.	Completed	China
CAR-GPC3 T cells in patients with refractory HCC	—	NCT 03146234	Relapsed or refractory HCC. GPC3 is expressed by IHC.	Recruiting	China
Glypican 3-specific CAR expressing T cells for HCC (GLYCAR)	I	NCT 02905188	Unresectable, recurrent, and/or metastatic HCC. GPC3-positive HCC.	Recruiting	United States
GPC3-T2-CAR-T Cells for Immunotherapy of Cancer With GPC3 Expression	I	NCT 03198546	Advanced HCC that expresses GPC3 protein.	Recruiting	China
Anti-GPC3 CAR-T for treating GPC3-positive advanced HCC	I & II	NCT 03084380	GPC3 is expressed by IHC. Patients with no ability to receive TACE combined with sorafenib.	Not yet recruiting	China
CAR-T cells targeting GPC3	I	NCT 03884751	Advanced HCC that is not suitable for surgery or local treatment, with no effective treatment after standard systemic therapies. GPC3 is expressed by IHC.	Not yet recruiting	China
4th generation CAR-T cells targeting GPC3	I	NCT 03980288	Advanced HCC that is not suitable for surgery or local treatment, with no effective treatment after standard systemic therapies. GPC3 is expressed by IHC.	Not yet recruiting	China
Clinical study of redirected autologous T cells with a CAR in patients with malignant tumors	—	NCT 03302403	HCC that cannot be eradicated by resection or ablation. GPC3 is expressed by IHC. Other malignancies.	Not yet recruiting	China

TACE: transcatheter arterial chemo-embolization.
